# Multi-resolution dimer models in heat baths with short-range and long-range interactions

**DOI:** 10.1098/rsfs.2018.0070

**Published:** 2019-04-19

**Authors:** Ravinda S. Gunaratne, Daniel B. Wilson, Mark B. Flegg, Radek Erban

**Affiliations:** 1 Mathematical Institute, University of Oxford, Radcliffe Observatory Quarter, Woodstock Road, Oxford OX2 6GG, UK; 2 School of Mathematical Sciences, Monash University, 9 Rainforest walk, Clayton campus, Victoria 3168, Australia

**Keywords:** molecular dynamics, generalized Langevin equation, intermolecular interactions, Langevin dynamics, multi-resolution modelling, multiscale methods

## Abstract

This work investigates multi-resolution methodologies for simulating dimer models. The solvent particles which make up the heat bath interact with the monomers of the dimer either through direct collisions (short-range) or through harmonic springs (long-range). Two types of multi-resolution methodologies are considered in detail: (a) describing parts of the solvent far away from the dimer by a coarser approach; (b) describing each monomer of the dimer by using a model with different level of resolution. These methodologies are then used to investigate the effect of a shared heat bath versus two uncoupled heat baths, one for each monomer. Furthermore, the validity of the multi-resolution methods is discussed by comparison to dynamics of macroscopic Langevin equations.

## 1. Introduction

Molecular dynamics (MD) approaches, based on the rules of classical mechanics, have been used to study the behaviour of complex biomolecules in biological applications [1,2]. They are written in terms of the positions and velocities of particles, representing either individual atoms or groups of atoms, describing parts of a biomolecule [3–6]. Inter-particle forces in MD models include combinations of short-range and long-range interactions [7,8]. In all-atom MD models, a common example of short-range forces are interactions described by the Lennard–Jones potential [9,10], while Coulomb forces provide an example of long-range forces [7]. Considering coarse-grained or caricature MD models, short-range interaction models include systems when particles only interact through direct collisions [11–14], while long-range interactions also include models where particles interact through harmonic springs [15,16]. Once the inter-particle interactions are specified, MD describes the time evolution of the model as a system of ordinary or stochastic differential equations for the positions of particles, which can also be subject to algebraic constraints, representing bonds between atoms or fixed internal structures of a biomolecule [2,17,18].

Biologically relevant simulations have to be done in aqueous solutions. A number of water models have been developed in the literature to use in all-atom MD simulations, including commonly used three-site (SPC/E, TIP3P) models [19,20]. In coarse-grained MD models, water is often treated with the same level of coarse-graining as other molecules in the system. For example, four water molecules are combined into a single coarse-grained water bead in the Martini model [3], while the Wat Four water model [6] uses four linked beads placed at the corners of a tetrahedron to collectively represent 11 water molecules. In this paper, we consider two theoretical heat baths which enable more analytical progress than solvent models based on all-atom or coarse-grained water models. In both cases, the convergence to the Langevin description of the solute particle can be established in a certain limit [11–16]. Our solute particle will also be treated with the same level of simplicity and described as a simple dimer molecule consisting of two monomers (beads) connected by a spring.

Multi-resolution (hybrid) methods use detailed and coarse-grained simulations in different parts of the simulation domain during the same dynamic simulation [21–24]. Such methods have been developed in different application areas and at different spatial and temporal scales in the literature, including dual-resolution approaches AdResS and H-AdResS for all-atom MD simulations [25–29], methods for coupling Brownian dynamics approaches with lattice-based stochastic reaction-diffusion models [30–32] or methods which make use of continuum mean-field equations for the macroscopic component of the simulation [33–35].

In some multi-resolution MD approaches, the region of high resolution moves together with the large microscopic structure of interest so that the high resolution model is always used for the whole considered structure, which can range in size from a single biomolecule (a protein or a DNA in solution [27,28]) to virus-like particles [36,37]. The structure of interest is placed in the centre of the simulation domain, and it is solvated using a detailed atomistic MD water in its immediate neighbourhood, which is coupled with a coarse-grained water description in the rest of the computational domain.

Another type of multi-resolution modelling is used for modelling of macromolecules where a detailed model of an important part of a macromolecule is coupled with a coarser model of the rest of the macromolecule. For example, atomistic detail of the active part of an enzyme has been coupled with a coarser model of the rest of the protein [38], different resolutions have been used in bead-spring modelling of DNA [39,40] or for modelling of polymer melts [41,42].

In this paper, we study both multi-resolution approaches using a simple dimer model consisting of two monomers (beads) connected by a spring. Similar models, where a macromolecule is described as several beads, representing parts of the simulated biomolecule, connected by springs, have been obtained in the literature using the method of ultra-coarse-graining [43]. Thus, our dimer model can be considered as a caricature of an ultra-coarse-grained model of a macromolecule. We study its behaviour in two theoretical heat baths. Our investigation focuses on multi-resolution (multiscale) descriptions of the solvent which can be described at the microscopic level of individual solvent molecules or at the macroscopic (dimer) level with the introduction of extrinsic random thermal forces on the monomers. We present models of the same dimer with various multi-resolution descriptions for the solvent and highlight the conditions and reasons, when and why, different model approximations of the solvent may be made in simulations.

Our paper is organized as follows. In §2, we introduce the macroscopic dimer model with a macroscopic description for solvent forces. This macroscopic model is fully described by Langevin equations. The Langevin macroscopic model is commonly used in simulation due to ease of implementation and analysis. We discuss in §2 the properties of this description with the intent to use these properties as benchmarks against which to compare microscopic and multi-resolution solvent models for the same dimer. Two theoretical microscopic approaches to model the solvent are introduced and studied through multi-resolution (simultaneous microscopic and macroscopic coupled) modelling in §§3 and 4. One of them is based on (very) short-range interactions, as heat bath particles only interact with the dimer on contact. The other one is at the opposite extreme, as it is based on (very) long-range interactions, where the heat bath is modelled as a system of many harmonic oscillators.

## 2. The dimer model

In this section, we will talk exclusively about the construction of the model for the dimer which will be used throughout this manuscript. In doing so, we describe the solvent at the macroscopic level as an extrinsically added random force. The result will be a set of Langevin equations. Throughout the manuscript, we will modify the treatment of the solvent forces at various scales and hybrid resolutions but the underlying dimer model will be the same.

We consider a model of a dimer which is described by positions of its two monomers, denoted by **X**_1_ = [*X*_1;1_, *X*_1;2_, *X*_1;3_] and **X**_2_ = [*X*_2;1_, *X*_2;2_, *X*_2;3_], respectively. Each monomer has the same mass, *M*. We denote by **R** the vector describing the separation between the monomers, i.e. **R** = **X**_2_ − **X**_1_, and by *R* its magnitude *R* = |**R**|. The interaction between monomers is given in terms of the potential Φ≡Φ(R):[0,∞)→R, which generates a force on each of the monomers with magnitude *Φ*′(*R*).

When the dimer is placed into a heat bath, there are additional forces on the two monomers caused by interactions with solvent molecules. The solvent forces can be modelled in a number of different ways and at various scales. In this manuscript, we consider two classes of models to describe the solvent–dimer interactions. The first, presented in §3, models the solvent as a bath of point particles which collide with the monomers and elastic collisions (short-range interactions) contribute to the generation of the forces. In the second case, described in §4, solvent molecules are point particles which oscillate around and interact at a distance (through long-range interactions) with the monomers. The solvent-dimer interactions are the sum of harmonic oscillatory forces acting on each of the monomers. Importantly, both descriptions under suitable assumptions lead to a macroscopic description of the dimer given by the following set of Langevin equations:

2.1 $$\text{d}X_{1}=V_{1}\;\text{d}t,$$

2.2 $$\text{d}V_{1}=\frac{\Phi^{\prime }(R)}{M}\;\frac{R}{R}\;\text{d}t-\gamma V_{1}\;\text{d}t+\gamma \sqrt{2D}\;\text{d}W_{1},$$

2.3 $$\text{d}X_{2}=V_{2}\;\text{d}t$$

2.4 $$\text{and}\;\text{d}V_{2}=-\frac{\Phi^{\prime }(R)}{M}\frac{R}{R}\;\text{d}t-\gamma V_{2}\;\text{d}t+\gamma \sqrt{2D}\;\text{d}W_{2},$$

where **V**_1_ = [*V*_1;1_, *V*_1;2_, *V*_1;3_] and **V**_2_ = [*V*_2;1_, *V*_2;2_, *V*_2;3_] are velocities of the first and second monomer, respectively, **W**_1_ and **W**_2_ are three-dimensional vectors of independent Wiener processes, *D* is a diffusion coefficient and *γ* is a friction coefficient, with dimension ${\left[\gamma ]=[\text{time}\right]}^{-1}$.

System (2.1)–(2.4) provides a macroscopic model of the dimer, which we compare with microscopic (or multi-resolution) MD simulations which explicitly model the solvent. Its validity for different MD models can be tested by comparing values of different dimer’s statistics at equilibrium, including its expected length *L*_*d*_, dimer velocity autocorrelation function *C*_*d*_(*τ*) and dimer diffusion constant *D*_*d*_, defined by

2.5 $$\begin{array}{rl} L_{d} & =\lim_{t\to \infty }\langle R\rangle ,\\ C_{d}(\tau ) & =\lim_{t\to \infty }\frac{1}{3}\langle \bar{V}(t+\tau )\cdot \bar{V}(t)\rangle \\ \text{and}\;\;D_{d} & =\lim_{t\to \infty }\frac{1}{6t}\left\langle {\left(\bar{X}(t)-\bar{X}(0)\right)}^{2}\right\rangle , \end{array}$$

where $\bar{X}=(X_{1}+X_{2})/2$ is the centre of mass of the dimer and $\bar{V}=(V_{1}+V_{2})/2$ is its velocity. These quantities can be obtained analytically for our macroscopic model (2.1)–(2.4) as follows. Adding equation (2.2) and equation (2.4) and noting that the sum of two independent Wiener processes is another Wiener process **W** with its variance equal to the sum of the variances of the original two processes, we obtain an Ornstein–Uhlenbeck process for $\bar{V}$ in the following form:

$$\text{d}\bar{V}=-\gamma \bar{V}\;\text{d}t+\gamma \sqrt{D}\;\text{d}W.$$

Therefore, we have

2.6 $$C_{d}(\tau )=\frac{D\gamma }{2}\exp[-\gamma \tau ].$$

Integrating over *τ*, we deduce

2.7 $$D_{d}=\int_{0}^{\infty }C_{d}(\tau )\;\text{d}\tau =\frac{D}{2}.$$

Taking the difference of equation (2.4) minus equation (2.2), implementing the over-damped assumption (where *γ* is large) and combining the independent Weiner processes into a single Weiner process **W** gives

$$\text{d}R=-\frac{2\Phi^{\prime }(R)}{M\gamma }\frac{R}{R}\;\text{d}t+2\sqrt{D}\;\text{d}W.$$

The stationary distribution corresponding to this process is proportional to exp [−*Φ*(*R*)/(*MDγ*)]. Normalizing, we find the distribution of dimer lengths equal to

$$\varrho (R)=\frac{\exp\left[-\frac{\Phi (R)}{MD\gamma }\right]}{4\pi \int_{0}^{\infty }r^{2}\exp\left[-\frac{\Phi (r)}{MD\gamma }\right]\;\text{d}r}.$$

In the simulations that follow in this manuscript, we shall be assuming the dimer potential acts like a linear spring with a rest length of ℓ_0_ and a spring constant of *k* between the two monomers. That is, we shall assume

2.8 $$\Phi (R)=\frac{k{\left(R-\ell_{0}\right)}^{2}}{2}.$$

Each monomer within the dimer is representing a half of a molecule of interest and the value of the spring constant indicates the flexibility in which the molecule can change its shape. In this paper, we consider the parameter regime where the spring constant *k* is sufficiently large so that the dimer has a well-defined structure. In the limit of large *k*, we have $\varepsilon =\;MD\gamma /(k\;\ell_{0}^{2})\ll 1$. Then, *L*_*d*_ can be calculated as

2.9 $$L_{d}\approx \ell_{0}\left(1+\frac{2MD\gamma }{k\;\ell_{0}^{2}}\right),$$

which is valid up to the first order in ɛ. In particular, the presence of heat baths extends the dimer from its rest length on average. In the following two sections, we study two theoretical MD models, where we use equations (2.6), (2.7) and (2.9) to compare the macroscopic theory with the results obtained by MD simulations.

## 3. Short-range interaction heat bath

We describe the two monomers as balls with radius *r*_0_ and mass *M* which interact with point solvent particles when they collide with them. In particular, this is a theoretical model of a (very) short-range interaction heat bath. Between collisions, monomers follow Newton’s Second Law of Motion in the form

3.1 $$M\frac{\text{d}V_{1}}{\text{d}t}=\Phi^{\prime }(R)\frac{R}{R}$$

and

3.2 $$M\frac{\text{d}V_{2}}{\text{d}t}=-\Phi^{\prime }(R)\;\frac{R}{R},$$

where, following our notation introduced in §2, positions and velocities of the monomers are denoted by **X**_*i*_ and **V**_*i*_, respectively, and **R** = **X**_2_ − **X**_1_.

Our short-range interaction heat bath is described in terms of positions $x_{i}^{\;j}$ and velocities $v_{i}^{\;j}$, of heat bath particles, where *i* = 1, 2 is the monomer number and *j* = 1, 2, 3, …, is the number of the heat bath particle. Notice that this formulation allows us to consider two important cases: (a) each monomer has its own heat bath; (b) a single heat bath is shared by both monomers. By comparing our simulation results in cases (a) and (b), we can explicitly investigate whether there are any significant hydrodynamic interactions between the monomers. In the case (b), we simplify our notation by describing particles of the single heat bath by

3.3 $$x^{\;j}=x_{1}^{\;j}=x_{2}^{\;j}\;\text{and}\;v^{\;j}=v_{1}^{\;j}=v_{2}^{\;j}.$$

In both cases (a) and (b), we assume that all heat bath particles have the same mass, *m*, and define (dimensionless) parameter *μ* by

$$\mu =\frac{M}{m}.$$

We are interested in the parameter regime where *μ* ≫ 1. Our MD model is based on elastic collisions of heavy monomers (balls with mass *M* and radius *r*_0_) with point heat bath particles with masses *m*. We assume that the collisions are without friction, then conservation of momentum and energy yields the following formulae for post-collision velocities [12]:

3.4 $${\tilde{V}}_{i}={\left[V_{i}\right]}^{∥}+\frac{\mu -1}{\mu +1}{\left[V_{i}\right]}^{⊥}+\frac{2}{\mu +1}{\left[v_{i}^{\;j}\right]}^{⊥}$$

and

3.5 $${\tilde{v}}_{i}^{\;j}={\left[v_{i}^{\;j}\right]}^{∥}+\frac{1-\mu }{\mu +1}{\left[v_{i}^{\;j}\right]}^{⊥}+\frac{2\mu }{\mu +1}{\left[V_{i}\right]}^{⊥},$$

where $v_{i}^{\;j}$ is the velocity of the heat bath particle which collided with the *i*th monomer, tildes denote post-collision velocities, superscripts ⊥ denote projections of velocities on the line through the centre of the monomer and the collision point on its surface, and superscripts ∥ denote tangential components.

Heat bath models based on elastic collisions (3.4) and (3.5) have been studied by a number of authors [11–14]. Consider a single monomer in infinite domain $R^{3}$, and let the heat bath consist of an infinite number of particles with positions distributed according to the spatial Poisson process with density

3.6 $$\lambda_{\mu }=\frac{3}{8r_{0}^{2}}\sqrt{\frac{(\mu +1)\gamma }{2\pi D}}.$$

This means that the number of points in a subset *Ω* of $R^{3}$ has its probability mass function given by the Poisson distribution with mean $\lambda_{\mu }|\Omega |$, where |*Ω*| is the volume of *Ω*. Let the velocities of the heat bath particles be distributed according to the Maxwell–Boltzmann distribution

3.7 $$f_{\mu }(v)=\frac{1}{\sigma_{\mu }^{3}{\left(2\pi \right)}^{3/2}}\exp\left[-\frac{v_{1}^{2}+v_{2}^{2}+v_{3}^{2}}{2\sigma_{\mu }^{2}}\right],$$

where **v** = [*v*_1_, *v*_2_, *v*_3_] and

3.8 $$\sigma_{\mu }=\sqrt{(\mu +1)\;D\;\gamma }.$$

Then the monomer’s behaviour is known to converge to the Langevin dynamics [12,14]. In particular, if we consider that each monomer has its own heat bath, we can show that the position and velocity of the monomers, **X**_*i*_ and **V**_*i*_, converge (in the sense of distributions) to the solution of (2.1)–(2.4) in the limit *μ* → ∞.

In reality, all beads representing a macromolecule exist within a single heat bath. Thus, we ask whether the correlations introduced by a bath of solvent which interacts with both monomers has a non-negligible affect on the equilibrium statistics of the dimer. Introducing such coupled heat baths for both short-range (in this section) and long-range (in §4) interactions, we study whether there is a significant difference between the one-bath and two-bath models as we vary ℓ_0_, the separation distance, introduced in equation (2.8). In order to study this problem, we make use of multi-resolution modelling.

### 3.1. Multi-resolution model using a co-moving frame

The solvent in the short-range heat bath interacts with the monomers of the dimer through direct contact. In order to simulate the model for long times, i.e. where the dimer has undergone a large excursion, the simulated domain must be vast as will be the number of solvent particles that must be modelled. We present a multi-resolution approach where we only model the solvent that is within the close vicinity of the dimer. We consider a co-moving cubic frame of length *L* that is centred at $X_{f}(t)$, which we here identify with the centre of mass of the dimer at time *t*, i.e.

3.9 $$X_{f}(t)=\bar{X}(t)=\frac{X_{1}(t)+X_{2}(t)}{2}.$$

Within this frame, we explicitly model the heat bath with solvent particles, i.e. they are simulated in the cubic box

3.10 $$X_{f}(t)+\left[-\frac{L}{2},\frac{L}{2}\right]\times \left[-\frac{L}{2},\frac{L}{2}\right]\times \left[-\frac{L}{2},\frac{L}{2}\right].$$

**Figure 1.**
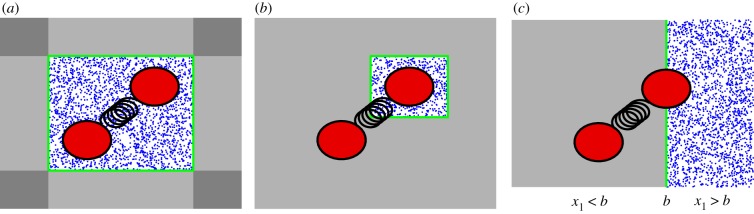
A diagrammatic representation of multi-resolution approaches for a dimer in a heat bath with short-range interactions. (*a*) Simulation of the whole dimer in a co-moving frame. The green box depicts the co-moving frame that is centred about the dimer. The blue dots correspond to solvent molecules that are explicitly modelled. Solvent molecules are not explicitly modelled in the external grey regions. (*b*) Simulation of one monomer in a co-moving frame. (*c*) Simulation with a fixed region of space where an MD model is explicitly used. A dimer molecule can move to the grey region where it is simulated using the Langevin description.

Externally, we model the heat bath as a continuum, where the particles are distributed according to the spatial Poisson process with density *λ*_*μ*_ given in (3.6) and the velocities are distributed according to *f*_*μ*_(**v**) given in (3.7), see figure 1*a* for a diagrammatic representation of the multi-resolution framework (drawn for clarity in two spatial dimensions, while all our simulations are three-dimensional). As the dimer moves around in $R^{3}$, the frame will move with it. In order for the multi-resolution model to capture, the full model where solvent particles are distributed in the entire domain, $R^{3}$, we need to introduce new solvent particles at the boundary of the frame.

Consider that time is discretized using small time step Δ*t*, i.e. if the current time is *t*, we want to calculate the state of the system at time *t* + Δ*t*. In our simulations of the multi-resolution model, we need the probability of introducing a particle at a boundary of the frame (3.10) in a timestep of length Δ*t* and subsequently the distribution of the position **x**_new_ and velocity **v**_new_ of the new solvent particle. For simplicity, we transform into the coordinate system of the co-moving frame which over an interval of length Δ*t* has velocity

3.11 $$V_{f}=\frac{X_{f}(t+\Delta t)-X_{f}(t)}{\Delta t}.$$

The frame is always translated to occupy the region [0, *L*]^3^. Thus, the velocities for the solvent particles in the new reference frame are given by $w^{\;j}=v^{\;j}-V_{f}$. We first calculate the density of particles that enter the frame via a particular boundary within a timestep of length Δ*t*. Take, as an illustrative example, the boundary face corresponding to {*x*_1_ = 0}. Consider particles which are in half-space $(-\infty ,0)\times R^{2}$ at time *t*. These particles have not yet been explicitly included in the simulation. Some of them will be in half-space $(0,\infty )\times R^{2}$ at time *t* + Δ*t*. Their density, *h*(*x*_1_), only depends on their first coordinate *x*_1_ ∈ (0, ∞). We can calculate *h*(*x*_1_) by integrating density (3.6) and (3.7) over solvent particles which are at $x_{1}^{\prime }\in (-\infty ,0)$ at time *t* and have the appropriate velocity to reach *x*_1_ ∈ (0, ∞) at time *t* + Δ*t*, namely as [14]

3.12 $$\begin{array}{rl} h(x_{1}) & =\int_{-\infty }^{0}\int_{R^{2}}\lambda_{\mu }f_{\mu }\left(\frac{x_{1}-x_{1}^{\prime }}{\Delta t}+V_{f;1},v_{2},v_{3}\right)\text{d}v_{2}\;\text{d}v_{3}\;\text{d}x_{1}^{\prime }\\ & =\frac{\lambda_{\mu }}{2}\;\text{erfc}\;\left(\frac{x_{1}+V_{f;1}\Delta t}{\sigma_{\mu }\Delta t\sqrt{2}}\right), \end{array}$$

where $V_{f;1}$ is the first component of the frame velocity and $\text{erfc}(z)=2/\sqrt{\pi }\int_{z}^{\infty }\exp(-s^{2})\;\text{d}s$ is the complementary error function. Integrating (3.12) over the domain (0, ∞) × [0, *L*] × [0, *L*] gives us the average number of particles that have entered the frame from the {*x*_1_ = 0} boundary in a time interval of length Δ*t* as

3.13 $$\begin{array}{rl} \;p_{\mathrm{in}} & =\int_{0}^{\infty }\int_{0}^{L}\int_{0}^{L}h(x_{1})\;\text{d}x_{3}\;\text{d}x_{2}\;\text{d}x_{1}\\ & =\lambda_{\mu }L^{2}\Delta t(\frac{\sigma_{\mu }}{\sqrt{2\pi }}\exp\left[-\frac{V_{f;1}^{2}}{2\sigma_{\mu }^{2}}\right]-\frac{V_{f;1}}{2}\text{erfc}\;\left[\frac{V_{f;1}}{\sigma_{\mu }\sqrt{2}}\right]). \end{array}$$

In our simulations, we choose a timestep small enough that $p_{\mathrm{in}}\ll 1$, we can therefore use $p_{\mathrm{in}}$ as the probability of introducing a new solvent particle. Let **z** = [*z*_1_; *z*_2_; *z*_3_] be the position of the new solvent particle in the coordinate system of the co-moving frame. Then coordinates *z*_2_ and *z*_3_ are uniformly distributed in (0, *L*) and the first coordinate can be sampled from the error function distribution

3.14 $$C_{1}\;\text{erfc}\;\left[\frac{z_{1}+V_{f;1}\Delta t}{\sigma_{\mu }\Delta t\sqrt{2}}\right],\;\;\text{for}\;z_{1}\in (0,\infty ),$$

where *C*_1_ is a normalizing constant. Then the position of the new solvent particle in the original coordinates is $x_{\mathrm{new}}=z+X_{f}(t+\Delta t)-[L/2,L/2,L/2]$. The velocity, **w**, of the new particle in the co-moving frame must have a first coordinate exceeding *z*_1_/Δ*t* in order to reach *z*_1_ in a time interval of length Δ*t*. Noting that $w=v_{\mathrm{new}}-V_{f}$ we write down the distribution of the velocity as the following truncated Gaussian distribution

3.15 $$C_{2}\;H(v_{1}\Delta t-(z_{1}+V_{f;1}\Delta t))f_{\mu }(v),$$

where *C*_2_ is a normalizing constant and H(⋅) is the Heaviside step function, satisfying *H*(*y*) = 1 for *y* ∈ [0, ∞) and *H*(*y*) = 0 otherwise. The position and velocity of solvent particles introduced at the other five faces can be done by symmetric modifications of the above distributions.

Random numbers from distributions (3.14) and (3.15) can be efficiently sampled using acceptance–rejection algorithms. We use an acceptance–rejection method for the truncated normal distribution (3.15) presented in the literature [44], while we sample random numbers from the distribution (3.14) using the acceptance–rejection algorithm presented in table 1. This is a generalization of the acceptance–rejection algorithm for sampling random numbers according to the distribution $\sqrt{\pi }\;\text{erfc}(z)$ previously used in simulations in the stationary frame [14]. In the case of the distribution (3.14), we need to sample random numbers according to the probability distribution

3.16 $$p(z;\beta )=C_{3}(\beta )\;\text{erfc}(z+\beta ),$$

where β∈R is a constant and *C*_3_(*β*) is the normalizing constant given by

3.17 $$C_{3}(\beta )=\frac{\sqrt{\pi }}{\exp[-\beta^{2}]-\sqrt{\pi }\beta \;\text{erfc}(\beta )}.$$

The algorithm in table 1 does this by generating an exponentially distributed random number *η*_3_ with mean *a*_1_(*β*), where

3.18 $$a_{1}(\beta )=\frac{\sqrt{\pi }}{2}\times \left\{ \begin{array}{cc} \text{erfc}(\beta )\exp(\beta^{2}), & \;\text{for}\;\beta \geq 0;\\ 1, & \;\text{for}\;\beta \leq 0. \end{array}\right.$$

To maximize the acceptance probability of this algorithm, we choose its second parameter, *a*_2_(*β*), as

3.19 $$a_{2}(\beta )=\left\{ \begin{array}{cc} \frac{1}{\text{erfc}(\beta )}, & \;\text{for}\;\beta \geq 0;\\ \exp\left(\frac{2\beta }{\sqrt{\pi }}\right), & \;\text{for}\;\beta \leq 0. \end{array}\right.$$

Then its acceptance probability is depending on *β* as

3.20 $$\frac{a_{2}(\beta )}{a_{1}(\beta )\;C_{3}(\beta )}.$$

**Figure 2.**
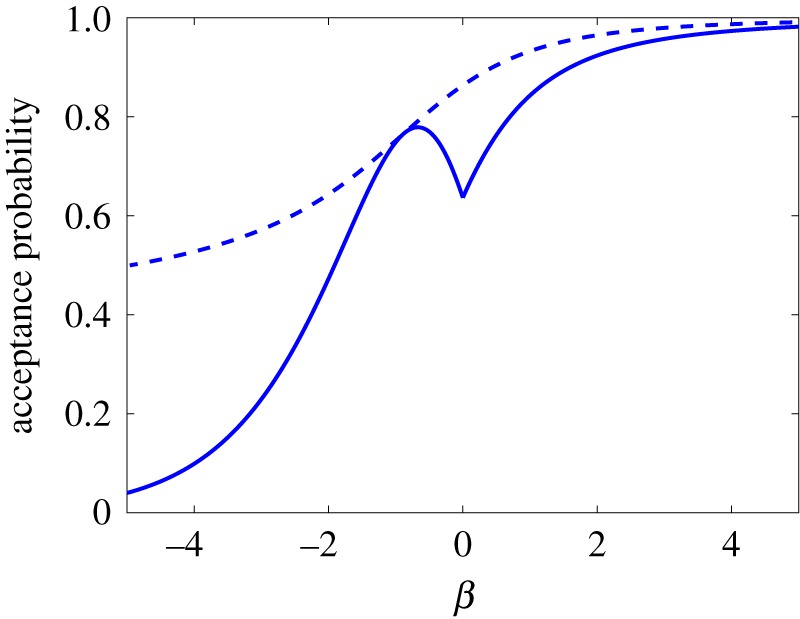
Plot of the acceptance probability (3.20) of the algorithm presented in table 1 for parameters given by (3.18) and (3.19) (solid line) compared with the acceptance probability (3.20) calculated for optimal choices of *a*_1_(*β*) and *a*_2_(*β*) for each parameter value *β*. (Online version in colour.)

We plot the acceptance probability (3.20) in figure 2 for our choices (3.18) and (3.19) of *a*_1_(*β*) and *a*_2_(*β*) as the solid line.

**Table 1.** Acceptance–rejection algorithm for sampling random numbers according to the probability distribution *p*(*z*; *β*) given by (3.16).

— Generate two random numbers *η*_1_ and *η*_2_ uniformly distributed in interval (0,1).
— Calculate *a*_1_(*β*) and *a*_2_(*β*) according to (3.18) and (3.19).
— Compute an exponentially distributed random number *η*_3_ by *η*_3_ = −*a*_1_(*β*) log (*η*_1_).
— If *η*_1_ *η*_2_ < *a*_2_(*β*) erfc(*η*_3_ + *β*), then choose *η*_3_ as a sample from the probability distribution (3.16). Otherwise, repeat the algorithm.

We observe that the acceptance probability (3.20) for *β* = 0 is equal to 2/π≈63.7%. This value can be improved [14] in the case of *β* = 0 to 86.3% provided that we choose *a*_1_ = 0.532 and *a*_2_ = 0.814. To obtain a similar improvement for all values of *β*, we could choose both *a*_1_(*β*) and *a*_2_(*β*) to maximize the acceptance probability (3.20), rather than postulating that *a*_1_(*β*) is given by the piecewise defined function (3.18) and optimizing *a*_2_(*β*) only. The acceptance probability (3.20) of the resulting algorithm (which would have *a*_1_(*β*) and *a*_2_(*β*) given by a lookup table, rather than by using formulae (3.18) and (3.19)) is plotted in figure 2 as the dashed line for comparison. However, in our illustrative simulations, we use the acceptance–rejection algorithm in table 1 with the values of *a*_1_(*β*) and *a*_2_(*β*) given by (3.18) and (3.19).

Comparing equations (3.16) and (3.14), we observe that we can sample random numbers from the distribution (3.14) by sampling random numbers from the distribution $p(z;V_{f;1}\Delta t)$ (using the acceptance-rejection algorithm in table 1 for $\beta =V_{f;1}\Delta t)$) and multiplying them by the factor $\sigma_{\mu }\Delta t\sqrt{2}$.

One iteration (i.e. an update of the state of the system from time *t* to time *t* + Δ*t*) of the multi-resolution simulation algorithm in the co-moving frame is given as algorithm [S1]–[S7] in table 2. It evolves the positions and velocities of both monomers together with the positions and velocities of *N*(*t*) solvent particles, where *N*(*t*) does depend on time *t*. To formulate algorithm [S1]–[S7], we assume that the timestep Δ*t* is chosen small enough so that at most one collision happens per iteration.

**Table 2.** One iteration of the multi-resolution simulation algorithm of the dimer in a co-moving frame.

[S1]	Update the positions of the solvent and the monomers by their ‘free-flight’ positions (3.21)–(3.22).
[S2]	If the ‘free-flight’ position (3.22) of a solvent particle lies within the radius of either of the monomers, reverse the trajectories of the solvent and the monomer by time *τ* < Δ*t* such that they are just touching. Calculate post-collision velocities by equations (3.4) and (3.5) and update their new positions by moving forward by time *τ*. Otherwise, each ‘free-flight’ position is accepted as the particle’s position at time *t* + Δ*t*.
[S3]	Update the velocities of the monomers by (3.23)–(3.24).
[S4]	Calculate the new centre of the co-moving frame, $X_{f}(t+\Delta t)$, by (3.9). Update *N*(*t*) by removing solvent particles which now lie outside of the frame (3.10) from the simulation.
[S5]	Calculate the velocity of the frame, $V_{f}$, over the interval [*t*, *t* + Δ*t*] by equation (3.11).
[S6]	Generate two random number *r*_1_ and *r*_2_ uniformly distributed in interval (0, 1). If $r<6p_{\mathrm{in}}$, then choose a side of the cube at random and generate proposed position $x_{\mathrm{new}}$ and velocity $v_{\mathrm{new}}$ of the new solvent particle according to distributions (3.14) and (3.15). If $r_{2}<h_{\mathrm{acc}}(x_{\mathrm{new}},v_{\mathrm{new}})$, then increase *N*(*t*) = 1 and initialize the new solvent particle at position $x_{\mathrm{new}}$ with velocity $v_{\mathrm{new}}$.
[S7]	Continue with step [S1] using time *t* = *t* + Δ*t*.

We initialize the two monomers with a separation distance ℓ_0_ and generate a Poisson number (with mean *λ*_*μ*_ *L*^3^) of solvent particles in our simulation domain, the cubic frame (3.10). The solvent particles are initially placed uniformly in the frame (3.10), where we remove particles overlapping with monomers (before we begin our simulation) to get the initial number, *N*(0), of simulated solvent particles. Their initial velocities are drawn from the Maxwell–Boltzmann distribution (3.7).

In step [S1], we update the system over the time interval (*t*, *t* + Δ*t*] using the ‘free-flight’ positions for each monomer and solvent particle, namely we use

3.21 $${\hat{X}}_{i}(t+\Delta t)=X_{i}(t)+V_{i}(t)\Delta t$$

and

3.22 $${\hat{x}}_{i}^{\;j}(t+\Delta t)=x_{i}^{\;j}(t)+v_{i}^{\;j}(t)\Delta t,$$

where *i* = 1, 2 is the monomer number and j=1,2, …,N(t), is the number of the heat bath particle. Since Δ*t* is chosen so small that only one collision happens during the time interval [*t*, *t* + Δ*t*), most of the ‘free-flight’ positions of solvent particles are accepted in step [S2] as their updated positions $x_{i}^{\;j}(t+\Delta t)$ and only the solvent particle colliding with a monomer is further updated.

In step [S3], we update the velocities of the monomers by solving (3.1) and (3.2) over one time step [*t*, *t* + Δ*t*]. We discretize (3.1) and (3.2) using the forward Euler method as follows:

3.23 $$V_{1}(t+\Delta t)={\tilde{V}}_{1}+\frac{\Phi^{\prime }(R)}{M}\frac{R}{R}\;\Delta t$$

and

3.24 $$V_{2}(t+\Delta t)={\tilde{V}}_{2}-\frac{\Phi^{\prime }(R)}{M}\frac{R}{R}\;\Delta t,$$

where ${\tilde{V}}_{i}$, for *i* = 1, 2, is either the post-collision velocity (if a collision happened in step [S2]) or is equal to **V**_*i*_(*t*). In steps [S4]–[S5], we update the position and velocity of the frame. We remove solvent particles which are outside of the simulation domain and update *N*(*t*) accordingly.

In step [S6], we use probability $p_{\mathrm{in}}$, given by (3.13), to check whether any solvent particle entered the simulation domain during the time interval (*t*, *t* + Δ*t*]. Since $p_{\mathrm{in}}$ is the probability of entering the domain through one of its six sides, we can, for time step Δ*t* small enough that $6p_{\mathrm{in}}\ll 1$, introduce at most one solvent particle through a randomly chosen side with probability $6p_{\mathrm{in}}$. The initial position and velocity of the introduced solvent particle are sampled according to distributions (3.14) and (3.15) or their symmetric modifications, taking into account through which side of the cubic frame (3.10) the particle entered the frame.

There is one little caveat in our derivation of $p_{\mathrm{in}}$. To derive equation (3.13), we integrated over the half-space $(-\infty ,0)\times R^{2}$, meaning that once we consider all six faces of the cubic frame (3.10) we have over-counted twice at the edges and three times at the corners (as it is highlighted with darker grey shading in our illustrative diagram in figure 1*a*). This will have negligible effect if we choose *L* sufficiently large. However, it can bias our simulation for values of *L* comparable with the monomer size *r*_0_ when Δ*t* is not sufficiently small as boundary effects become more pronounced. To compensate for this effect, we consider the sampled position, $x_{\mathrm{new}}$ and velocity $v_{\mathrm{new}}$ of the new incoming particle at time *t* + Δ*t* and calculate its previous position at time *t* by

$$y=x_{\mathrm{new}}-v_{\mathrm{new}}\;\Delta t.$$

If **y** is in the regions which were counted twice or three times in our derivation, we reject the proposed introduction of the new solvent particle with the corresponding probability. Namely, we use the acceptance probability in step [S6] given by

$$h_{\mathrm{acc}}(x_{\mathrm{new}},v_{\mathrm{new}})=\left\{ \begin{array}{cc} 1, & \;\text{for}\;y-X_{f}(t)\in Y_{1};\\ \frac{1}{2}, & \;\text{for}\;y-X_{f}(t)\in Y_{2};\\ \frac{1}{3}, & \;\text{for}\;y-X_{f}(t)\in Y_{3}, \end{array}\right.$$

where $Y_{\;j}\subset R^{3}$ is the region of the space which consists of points which have exactly *j* of their coordinates outside of the interval [−*L*/2, *L*/2]. For example, in our two-dimensional diagrammatic representation in figure 1*a*, the lighter grey shading corresponds to region $Y_{1}$ while the darker grey shading corresponds to region $Y_{2}$.

**Figure 3.**
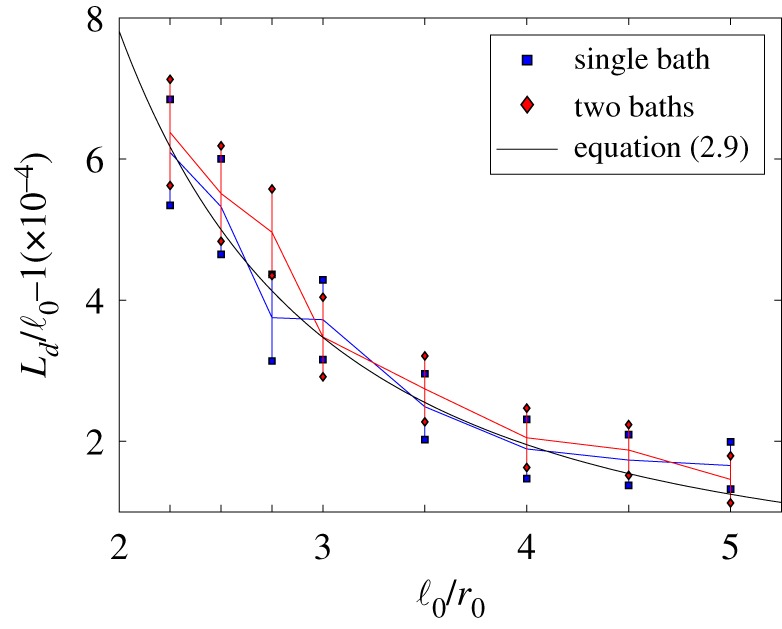
The extension of the average length of a dimer from its separation distance ℓ_0_. The equilibrium data for each model was collected from a long-time simulation of length 100 dimensionless time units where Δ*t* = 10^−6^ and the monomers were initially placed with separation ℓ_0_. The values of *α* = ℓ_0_/*r*_0_ presented are {2.25, 2.5, 2.75, 3, 3.5, 4, 4.5, 5}. The parameters used are *r*_0_ = 0.08, *γ* = 10, *D* = 1, *μ* = 10^3^ and *k* = 10^6^. In the one-bath case, we use *L* = 0.72 for the frame (3.10) enclosing the whole dimer, and for the two-bath case, we use *L* = 0.32 for each monomer frame.

In our illustrative simulations, we use algorithm [S1]–[S7] from table 2 together with parameter values *r*_0_ = 0.08, *γ* = 10, *D* = 1, *μ* = 10^3^, *k* = 10^6^ and *L* = 0.72 for the one-bath case. In figure 3, we compare simulation results of the average length of the dimer at equilibrium, *L*_*d*_, for the one-bath and two-bath models. Since the two-bath case uses uncoupled heat baths, we can further improve the efficiency of our algorithm by centring the co-moving frame corresponding to each heat bath on the corresponding monomer, i.e. we use $X_{f}(t)=X_{i}(t)$ for the heat bath corresponding to the *i*th monomer in step [S4] (instead of the centre of mass (3.9)) and choose smaller value of *L* in the two-bath case, namely *L* = 0.32. In both one-bath and two-bath models, the solvent particles are distributed according to the spatial Poisson process with density $\lambda_{\mu }$ given by (3.6). The velocities are distributed according to the Maxwell–Boltzmann distribution *f*_*μ*_(**v**) given by (3.7). We note that in the two-bath case, our model converges to the Langevin dynamics (2.1)–(2.4) as *μ* → ∞. This allows us to attribute any changes between the one-bath case and the Langevin model to the correlations induced by sharing a heat bath. The asymptotic analytic result obtained for the Langevin model, equation (2.9), is plotted as the black solid line for comparison.

In figure 3, we set the separation distance to be ℓ_0_ = *αr*_0_ where *α* ≥ 2, such that at this distance apart the monomers are not overlapping. The plot shows the two-sided 99% confidence intervals for (*L*_*d*_ − ℓ_0_)/ℓ_0_ for *α* ∈ {2.25, 2.5, 2.75, 3, 3.5, 4, 4.5, 5}. Firstly, we note that *L*_*d*_ > ℓ_0_ in each of the models as predicted in (2.9). There seems to be reasonable correspondence between the one- and two-bath models, with the confidence intervals overlapping. This suggests that the correlations we lose by approximating a larger co-moving frame around both monomers with two smaller dedicated frames around each monomer are negligible, allowing us to increase efficiency without biasing our overall results. In the next section, we build on this observation and present a multi-resolution framework which replaces one of the smaller dedicated frames by a coarser model of the heat bath, written in terms of the Langevin dynamics.

### 3.2. Monomers with different resolution

As the length of a polymer (i.e. numbers of monomers) increases, a model incorporating solvent particles around each of the monomers becomes increasingly computationally expensive. However, a fully coarse-grained Langevin model of a polymer such as the Rouse model [39] can lack the required level of detail. Thus, some multi-resolution approaches for simulating macromolecules only model an important (small) part of a macromolecule using a detailed modelling approach [38–42]. In our case, we can mimic such methodologies by modelling the first monomer with explicit solvent with a heat bath of physical molecules, while the second monomer is modelled using the Langevin equations (2.3) and (2.4). Such a multi-resolution approach is schematically shown in figure 1*b*. To simulate this model, we use a co-moving frame, given by equation (3.10), which is centred around the first monomer, i.e. $X_{f}(t)=X_{1}(t)$.

One iteration of the algorithm is presented as algorithm [M1]–[M5] in table 3. To begin, we initialize the particle positions and velocities in the similar way as in the case of algorithm [S1]–[S7], with the only difference that the cubic frame (3.10) is now centred around the first monomer. steps [M1] and [M2] are directly equivalent to steps [S1] and [S2]. In step [M3], we update the position and velocity of the second monomer by

3.25 $$\begin{array}{rl} V_{2}(t+\Delta t) & =V_{2}(t)-\left(\frac{\Phi^{\prime }(R)}{M}\frac{R}{R}+\gamma V_{2}(t)\right)\Delta t+\gamma \sqrt{2D\Delta t}\xi , \end{array}$$

where *ξ* is sampled from the normal distribution with zero mean and unit variance. That is, we have replaced the heat bath of the second monomer by solving the corresponding Langevin equation (2.1)–(2.4) using the standard Euler–Maruyama integrator. There have been other schemes developed in the literature for discretizing the Langevin equation such as van Gunsteren & Berendsen [45] and the Langevin Impulse integrators, which capture the Langevin dynamics more accurately especially in the presence of forces, such as the spring force between the monomers [46]. Another option would be to consider the BBK integrator [47], which we use in §4.1, where we present a multi-resolution algorithm for the long-range interaction heat bath model and discretize the Langevin equation using a combination of the velocity Verlet and Euler–Maruyama integrators, see equations (4.11)–(4.15). An additional approach is the Verlet scheme [48] that approximates the velocity using a central difference discretization rather than the forward difference approach used in the Euler–Maruyama method, or Runge–Kutta methods [49], which could further reduce the error of the multi-resolution simulations.

**Table 3.** One iteration of the multi-resolution simulation algorithm of the dimer in the heat bath with short-range interactions, where the second monomer is simulated by the Langevin dynamics.

[M1]	Update the positions of the solvent and the monomers by their ‘free-flight’ positions (3.21) and (3.22).
[M2]	If the ‘free-flight’ position (3.22) of a solvent particle lies within the radius of the first monomer, reverse the trajectories of the solvent and the monomer by time *τ* < Δ*t* such that they are just touching. Calculate post-collision velocities by equations (3.4) and (3.5) for *i* = 1 and update their new positions by moving forward by time *τ*. Otherwise, each ‘free-flight’ position is accepted as the particle’s position at time *t* + Δ*t*.
[M3]	Update the velocity of the first monomer by (3.23) and the velocity of the second monomer by (3.25).
[M4]	Calculate the new centre of the co-moving frame as $X_{f}(t+\Delta t)=X_{1}(t)$. Update *N*(*t*) by removing solvent particles which now lie outside of the frame (3.10) from the simulation. Use steps [S5]–[S6] from the algorithm in table 2 to introduce new solvent particles into the co-moving frame (3.10).
[M5]	Continue with step [M1] using time *t* = *t* + Δ*t*.

**Figure 4.**
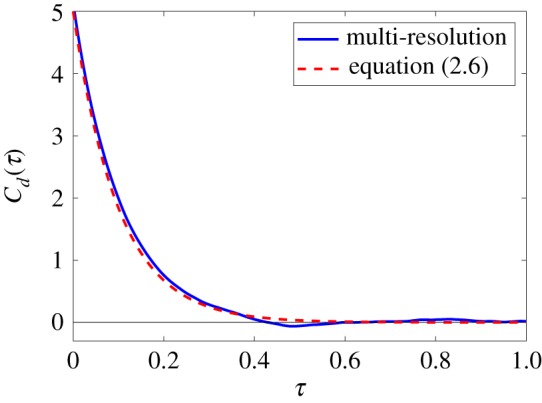
The velocity autocorrelation function for the multi-resolution model (solid line) for short-range interactions. The function is estimated from long time simulation over dimensionless time of 500 time units. It is compared with the result for the Langevin description of the whole dimer, given by equation (2.6) (dashed line). The parameters are *r*_0_ = 0.08, *γ* = 10, *D* = 1, *μ* = 10^3^, *k* = 10^6^, ℓ_0_ = 4*r*_0_, Δ*t* = 10^−6^ and *L* = 0.32. (Online version in colour.)

In order to compare simulations of the multi-resolution model with simulations of the Langevin model (2.1)–(2.4), we use the velocity autocorrelation function of the dimer, *C*_*d*_(*τ*), given by equation (2.5). It has been analytically calculated for the Langevin description in equation (2.6). In figure 4, we present numerical estimates of the velocity autocorrelation function of the multi-resolution model from long time simulation data, using definition (2.5).

Our results compare well with the theoretical result for the Langevin model, though it seems like there is a slightly raised value for *C*_*d*_(0). Using (2.7), we can estimate the diffusion constant of the dimer *D*_*d*_ by numerically integrating the velocity auto-correlation function in interval [0, 1]. We obtain *D*_*d*_ ≈ 0.529, while its theoretical value for the dimer model is given in equation (2.7) as *D*/2 = 0.5. Another approach is to fit the exponential function, in the form equation (2.6), to the computational result presented in figure 4. In this way, the values of both *D* and *γ* can be estimated simultaneously. We found that *D* ≈ 1.0714, which is higher than our parameter value *D* = 1, and *γ* ≈ 9.6064, which is lower than *γ* = 10 used in our simulations. This could suggest that the value of $\lambda_{\mu }$ is too low or that of *σ*_*μ*_ is too high in our simulations. However, when these quantities are measured during the simulations we do not observe any deviation. This suggests that, rather than our sampling methods, there are small errors introduced by our implementation of the moving frame, or more profound boundary effects introduced by the small size of the frame. A potential problem in the implementation of the co-moving frame, is that solvent particles that leave the frame never return. For a stationary frame, this is valid as the monomer cannot interact with a particle that leaves. However, for a co-moving small frame centred about the monomer, a solvent particle could leave the frame and return at a later time in the simulation. This is not taken into account in the presented algorithms.

## 4. Long-range interaction heat bath

Coarse-grained models of molecular systems can be written in terms of beads interacting through coarse-grained force fields. Each bead represents a collection of atoms and a coarse-grained potential energy can be constructed from detailed all-atom MD. Such an approach can usually provide a good description of equilibrium properties of molecular systems, but it does not necessarily lead to correct dynamics if the time evolution of the system is solely based on the Hamiltonian dynamics corresponding to the coarse-grained potential energy surface [50]. Dynamical behaviour can be corrected by introducing additional degrees for freedom (fictitious particles) interacting with each coarse-grained bead [50–52]. Fictitious particles can then be subject to suitable friction and noise terms to correct the dynamics.

Considering our dimer molecule model as an example of a coarse-grained molecule, written in terms of two coarse-grained beads (monomers) interacting through coarse-grained potential energy (2.8), then each monomer could be coupled with one or several fictitious particles interacting with the monomer through a suitable harmonic spring term [50,51]. Our long-range interaction heat bath is based on this approach, by assuming that the *i*th monomer, *i* = 1, 2, is coupled with *N*_*i*_ harmonic oscillators, in a manner similar to well-known theoretical heat bath models [15,16]. Then equations (3.1) and (3.2), expressing Newton’s Second Law of Motion, include additional terms as follows [15]:

4.1 $$M\frac{\text{d}V_{1}}{\text{d}t}=\Phi^{\prime }(R)\;\frac{R}{R}+\sum_{\;j=1}^{N_{1}}k_{1,j}\alpha_{1,j}(x_{1}^{\;j}-\alpha_{1,j}X_{1})$$

and

4.2 $$M\frac{\text{d}V_{2}}{\text{d}t}=-\Phi^{\prime }(R)\frac{R}{R}+\sum_{\;j=1}^{N_{2}}k_{2,j}\alpha_{2,j}(x_{2}^{\;j}-\alpha_{2,j}X_{2}),$$

where $x_{i}^{\;j}$ is the position of the *j*th solvent particle which interacts with the *i*th monomer through a harmonic spring with spring constant *k*_*i*,*j*_ and interaction constants *α*_*i*,*j*_, $j=1,\;2,\;\ldots ,N_{i}$, *i* = 1, 2. Equations (4.1) and (4.2) are coupled with the evolution equations for solvent particles. We assume that $v_{i}^{\;j}$ is the velocity of the *j*th solvent particle interacting with the *i*th monomer. Moreover, we assume that all oscillators have the same mass, *m*. Using Newton’s Second Law of Motion, we get the following evolution equations for the heat bath oscillators:

4.3 $$\frac{\text{d}x_{i}^{\;j}}{\text{d}t}=v_{i}^{\;j}$$

and

4.4 $$m\frac{\text{d}v_{i}^{\;j}}{\text{d}t}=-k_{i,j}(x_{i}^{\;j}-\alpha_{i,j}X_{i}),$$

for $j=1,2,\;\ldots ,\;N_{i}$ and *i* = 1, 2. Unlike in some fictitious particle models [50–52], we do not include friction and random forces into equation (4.4) for solvent, because we assume that we explicitly model all solvent particles, i.e. *N*_1_ and *N*_2_ are considered to satisfy *N*_1_ ≫ 1 and *N*_2_ ≫ 1. We are therefore working ‘close’ to the limit *N*_1_ → ∞ and *N*_2_ → ∞, in which we can get the convergence of our long-range interaction heat bath to the Langevin dynamics as discussed below. In practice, it is impossible to include all solvent molecules in simulations and friction and noise terms are still included to control temperature of the simulated system [2,53]. We can solve the solvent equations of motion (4.3) and (4.4) to give [2,54]

$$\begin{array}{rl} x_{i}^{\;j} & =x_{i}^{\;j}(0)\cos(\omega_{i,j}t)+\frac{v_{i}^{\;j}(0)}{\omega_{i,j}}\sin(\omega_{i,j}t)\\ & +\alpha_{i,j}\omega_{i,j}\int_{0}^{t}\sin(\omega_{i,j}(t-\tau ))\;X_{i}(\tau )\;\text{d}\tau , \end{array}$$

where $x_{i}^{\;j}(0)$ is the initial position of the *j*th heat bath particle corresponding to the *i*th monomer, $v_{i}^{\;j}(0)$ is its initial velocity and *ω*_*i*,*j*_ = (*k*_*i*,*j*_/*m*)^1/2^ is its frequency. Substituting for $x_{1}^{\;j}$ and $x_{2}^{\;j}$ in dimer’s equations of motion (4.1) and (4.2), we obtain the following coupled system of generalized Langevin equations:

4.5 $$M\frac{\text{d}V_{1}}{\text{d}t}=\Phi^{\prime }(R)\frac{R}{R}-\int_{0}^{t}\kappa_{1}(\tau )V_{1}(t-\tau )\;\text{d}\tau +\xi_{1}$$

and

4.6 $$M\frac{\text{d}V_{2}}{\text{d}t}=-\Phi^{\prime }(R)\;\frac{R}{R}-\int_{0}^{t}\kappa_{2}(\tau )\;V_{2}(t-\tau )\;\text{d}\tau +\xi_{2},$$

where the friction kernel *κ*_*i*_(*τ*) and noise term *ξ*_*i*_ ≡ *ξ*_*i*_(*t*) = [*ξ*_*i*;1_, *ξ*_*i*;2_, *ξ*_*i*;3_] are given by

$$\begin{array}{rl} \kappa_{i}(\tau ) & =m\sum_{\;j=1}^{N_{i}}\alpha_{i,j}^{2}\omega_{i,j}^{2}\cos(\omega_{i,j}\tau )\\ \text{and}\;\xi_{i}(t) & =m\sum_{\;j=1}^{N_{i}}x_{i}^{\;j}(0)\alpha_{i,j}\;\omega_{i,j}^{2}\cos(\omega_{i,j}t)\\ & +v_{i}^{\;j}(0)\alpha_{i,j}\omega_{i,j}\sin(\omega_{i,j}t), \end{array}$$

for *i* = 1, 2. We assume that initial positions and velocities of solvent oscillators, $x_{i}^{\;j}(0)$ and $v_{i}^{\;j}(0)$, are both independently sampled according to their equilibrium distributions. Then noise autocorrelation function is given by the generalized fluctuation–dissipation theorem

$$\lim_{t\to \infty }\langle \xi_{i;j}(t)\xi_{i;n}(t-\tau )\rangle =2k_{B}T\delta_{\;j,n}\kappa_{i}(\tau ),$$

where *k*_*B*_ is the Boltzmann constant and *T* is the absolute temperature. Next, we assume that the frequencies *ω*_*i*,*j*_ are sampled from a (continuous) exponential distribution with mean $\bar{\omega }$ and we set our interaction constants equal to

4.7 $$\alpha_{i,\;j}=\frac{1}{\omega_{i,\;j}}\sqrt{\frac{2\;\gamma \;\bar{\omega }}{N_{i}\;m\;\pi }}\;,$$

where *γ* > 0 is the friction constant used in equations (2.2) and (2.4). Then friction kernel (4.7) becomes

$$\kappa_{i}(\tau )=\frac{2\gamma \bar{\omega }}{\pi }\frac{1}{N_{i}}\sum_{\;j=1}^{N_{i}}\cos(\omega_{i,j}\tau ).$$

Passing to the limit *N*_*i*_ → ∞ allows us to consider the above summation as a continuous integral over the distribution of oscillator frequencies, with both friction kernels *κ*_1_(*τ*) and *κ*_2_(*τ*) converging to the same friction kernel [54]

4.8 $$\begin{array}{rl} \kappa (\tau ) & =\frac{2\gamma }{\pi }\int_{0}^{\infty }\cos(\omega \tau )\exp\left(-\frac{\omega }{\bar{\omega }}\right)\;\text{d}\omega \\ & =\frac{2\gamma }{\pi }\frac{\bar{\omega }}{{\bar{\omega }}^{2}\tau^{2}+1}. \end{array}$$

Then $\int_{0}^{\infty }\kappa (\tau )\;\text{d}\tau =\gamma .$ Moreover, we can define the limiting friction kernel by

$$\kappa_{\infty }(\tau )=\lim_{\bar{\omega }\to \infty }\kappa (\tau ),$$

which, for our choice of oscillators’ frequencies and interaction terms (4.7), satisfies *κ*_∞_(*τ*) = 0 for *τ* > 0 and *κ*_∞_(0) = ∞. Thus, the limiting kernel is a multiple of the Dirac delta function. Therefore, the position and velocity of the monomers, **X**_*i*_ and **V**_*i*_, converge to the solution of (2.1)–(2.4) in the limit $\bar{\omega }\to \infty$, provided that each monomer has its own separate heat bath. Moreover, we obtain the Einstein–Smoluchowski relation for the diffusion constant of the monomer as *D* = *k*_*B*_*T*/(*γM*).

As in §3, we have explained our MD model of the dimer using the case where each monomer has its own heat bath. We now turn our attention to the case when monomers share their heat bath. This has been studied in the case of the short-range interaction MD model in §3.1 with the help of multi-resolution modelling in a co-moving frame, as schematically shown in figure 1*a*. In the case of long-range interactions, a co-moving frame is less straightforward to implement because we need to take into account that particles outside of the simulated box do exert (long-range) forces on particles in our simulation domain. Some multi-resolution techniques in the literature solve this problem by introducing suitable overlap (bridging, blending) regions [51,55–57], where molecules which are near the simulation domain exert some partial forces on the simulated molecules.

**Figure 5.**
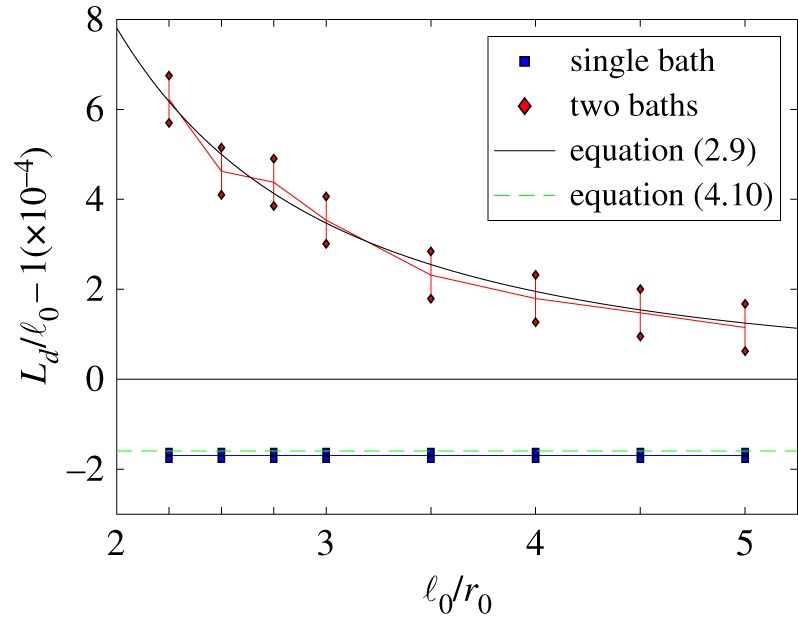
The extension of the average length of a dimer from its separation distance ℓ_0_ for long-range interaction heat bath models. The values of parameters are the same as in figure 3, together with $\bar{\omega }=100$, *N*_1_ = *N*_2_ = *N* = 10^4^, *M* = 1 and *m* = 10^−3^, which give the same value of *μ* = *M*/*m* as used in figure 3. The simulations for the single heat bath case use parameter choice (4.9) with $\alpha_{\;j}^{2}=\gamma \bar{\omega }/(N\pi k_{\;j})$, $k_{\;j}=m\omega_{\;j}^{2}/2$ and *ω*_*j*_ sampled according to the exponential distribution with mean $\bar{\omega }$, confirming result given in equation (4.10) (green dashed line). The results for the two heat bath case are compared with the result obtained for the Langevin model in equation (2.9) (black solid line).

In what follows, we do not truncate the simulated domain, but we consider a different multi-resolution approach in §4.1. Before then we discuss results comparable to figure 3, i.e. we compare simulations with a single heat bath and two heat baths for the case of our long-range interaction MD model. The results are presented in figure 5, where we use the same values of ℓ_0_ as in figure 3, expressed as *α*-multiples of *r*_0_, although our long-range interaction model does not make use of parameter *r*_0_. The value of *L*_*d*_ is for each value of ℓ_0_ calculated from a long simulation over 200 dimensionless time units, where the first 100 time units are used to equilibrate the system, while the second half of each simulation is used to compute *L*_*d*_. To initialize this model, we start with monomers separated by the rest length ℓ_0_ and sample oscillators’ frequencies, *ω*_*i*,*j*_, according to the exponential distribution with mean $\bar{\omega }$. Their positions and velocities are sampled from the Maxwell–Boltzmann distribution. For the two-bath model, each dimer particle is separately initialized with its own set of oscillators around their respective positions in space.

In figure 5, we observe that in the case of the two-bath model we obtain results which match well with equation (2.9) for our parameter values. These results are also directly comparable with the results obtained for the two-bath case in figure 3. The situation is more complicated in the case of simulations with a single heat bath with *N* oscillators. Then, using notation (3.3), we can rewrite (4.1) and (4.2) as

$$M\frac{\text{d}V_{i}}{\text{d}t}={\left(-1\right)}^{i+1}\Phi^{\prime }(R)\frac{R}{R}+\sum_{\;j=1}^{N}k_{i,j}\alpha_{i,j}(x^{\;j}-\alpha_{i,j}X_{i}),$$

for *i* = 1, 2, where the heat bath evolution equation (4.4) includes terms corresponding to both monomers

$$m\frac{\text{d}v^{\;j}}{\text{d}t}=-k_{1,j}(x^{\;j}-\alpha_{1,j}X_{1})-k_{2,j}(x^{\;j}-\alpha_{2,j}X_{2}),$$

for j=1,2, …, N. Our results will then depend how we choose parameters *k*_*i*,*j*_ and *α*_*i*,*j*_. For example, if we choose *k*_*i*,*j*_ and *α*_*i*,*j*_ to be the same for both monomeres, i.e.

4.9 $$k_{1,j}=k_{2,j}=k_{\;j}\;\text{and}\;\alpha_{1,j}=\alpha_{2,j}=\alpha_{\;j},$$

for j=1,2, …, N, then the oscillating frequency of the *j*th heat bath oscillator is $\omega_{\;j}=\sqrt{2k_{\;j}/m}$ and we can subtract the evolution equations for monomers to obtain

$$M\frac{{\text{d}}^{2}R}{\text{d}t^{2}}=-2\Phi^{\prime }(R)\frac{R}{R}-\sum_{\;j=1}^{N}k_{\;j}\alpha_{\;j}^{2}R.$$

This equation does not contain any heat bath variables. Using (4.7) to select *α*_*j*_, i.e. using $\alpha_{\;j}^{2}=2\gamma \bar{\omega }/(Nm\pi \omega_{\;j}^{2})=\gamma \;\bar{\omega }/(N\pi k_{\;j})$, we get

$$M\frac{{\text{d}}^{2}R}{\text{d}t^{2}}=-2\Phi^{\prime }(R)\frac{R}{R}+\frac{\gamma \bar{\omega }}{\pi }R.$$

Using potential (2.8), we conclude that we effectively obtain a shorter rest length of the spring which gives the following approximation:

4.10 $$L_{d}\approx \frac{2\;k\;\pi \;\ell_{0}}{2\;k\;\pi +\gamma \;\bar{\omega }}.$$

This result is plotted in figure 5 together with results obtained by illustrative simulations. We use a long-time simulation of length 200 dimensionless time units, with monomers initially placed at separation ℓ_0_ and averaging over the second half of the simulation (of length 100 dimensionless time units) to obtain the presented values of dimer’s expected length *L*_*d*_.

In figure 5, we observe that the average dimer length, *L*_*d*_, during our single heat bath simulations is smaller than the natural length of the spring, ℓ_0_. However, this conclusion is only a consequence of our choice of parameters (4.9). An opposite phenomenon can be observed in simulations for other parameter regimes. For example, if we divide our oscillators into two groups consisting of *N*_1_ and *N*_2_ oscillators, i.e. *N* = *N*_1_ + *N*_2_, and choose our parameters *k*_*i*,*j*_ and *α*_*i*,*j*_ such that

$$\begin{array}{rl} & k_{2,j}=0,\;\;\text{for}\;j=1,2,\;\ldots ,\;N_{1},\\ \text{and}\; & k_{1,j}=0,\;\;\text{for}\;j=N_{1}+1,N_{1}+2,\;\ldots ,\;N, \end{array}\;$$

then our ‘one-bath’ case is effectively equal to the two-bath case for which we have the result given in equation (2.9) presented in figure 5. In particular, depending on our choices of *k*_*i*,*j*_ and *α*_*i*,*j*_, the single heat bath case can both increase or decrease the average length of the dimer.

### 4.1. Multi-resolution modelling of dimer

In figure 1*b*, we use our dimer example to illustrate a multi-resolution approach which models a part of a molecule using a detailed MD approach, while using a coarser description of the rest of the molecule. Here, in the same manner as carried out for our short-range model in §3.2, we illustrate such a multi-resolution approach using our long-range interaction MD model. We use the Langevin model (2.1)–(2.4) to coarse-grain one of the monomers, while the other monomer is modelled in detail using the MD model with its heat bath described by harmonic oscillators (4.3) and (4.4). As in figure 4, we again calculate numerical estimates for the velocity autocorrelation function, *C*_*d*_(*τ*) in equation (2.5), from long time simulations of the dimer after equilibrium has been reached.

The pseudo-code of one iteration our multi-resolution algorithm is presented as algorithm [L1]–[L5] in table 4. algorithm [L1]–[L5] is based on the velocity Verlet integrator, where both monomers are updated by

4.11 $$V_{i}\left(t+\frac{1}{2}\Delta t\right)=V_{i}(t)+\frac{1}{2}A_{i}(t)\Delta t,$$

4.12 $$X_{i}(t+\Delta t)=X_{i}(t)+V_{i}\left(t+\frac{1}{2}\Delta t\right)\Delta t$$

4.13 $$\text{and}\;V_{i}(t+\Delta t)=V_{i}\left(t+\frac{1}{2}\Delta t\right)+\frac{1}{2}A_{i}(t+\Delta t)\Delta t,$$

where **A**_*i*_, for *i* = 1, 2, is the acceleration of the corresponding monomer. For the first monomer, its acceleration **A**_1_ is defined as the right-hand side of equation (4.1) divided by *M*, i.e.

4.14 $$A_{1}=\frac{\Phi^{\prime }(R)}{M}\;\frac{R}{R}+\frac{1}{M}\sum_{\;j=1}^{N_{1}}k_{1,j}\alpha_{1,j}(x_{1}^{\;j}-\alpha_{1,j}X_{1}).$$

For the second monomer, we use the BBK integrator [47], i.e. we define its acceleration as

4.15 $$A_{2}=-\frac{\Phi^{\prime }(R)}{M}\frac{R}{R}-\gamma V_{2}+\gamma \sqrt{\frac{2D}{\Delta t}}\xi ,$$

where *ξ* is sampled from the normal distribution with zero mean and unit variance. The corresponding solvent oscillator integrator is identical to the scheme (4.11)–(4.13), with **X**_1_, **V**_1_ and **A**_1_ replaced by **x**^*j*^, **v**^*j*^ and **a**^*j*^, respectively, where acceleration **a**^*j*^ is defined as the right-hand side of equation (4.4) divided by *m*, i.e.

4.16 $$a^{\;j}=-\frac{k_{i,j}}{m}(x_{i}^{\;j}-\alpha_{i,j}X_{i}).$$

**Figure 6.**
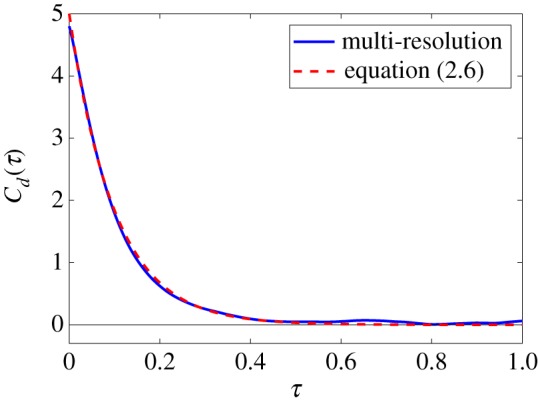
The velocity autocorrelation function for the multi-resolution model (solid line) for long-range interactions, estimated from long time simulation over dimensionless time of 10^3^ dimensionless time units. It is compared with the result for the Langevin description of the whole dimer, given by equation (2.6) (dashed line). The parameters are the same as in figure 4, namely *γ* = 10, *D* = 1, *k* = 10^6^, *M* = 1, *m* = 10^−3^, ℓ_0_ = 0.32, together with $\bar{\omega }=100$ and *N*_1_ = 10^5^. (Online version in colour.)

The results obtained by algorithm [L1]–[L5] are compared with analytic results given by equation (2.6) for the Langevin model in figure 6. We see that there is a good correspondence between these, suggesting that the value $\bar{\omega }=100$ is large enough to create an accurate Dirac delta approximation from the kernel function (4.8), along with having a large enough number of oscillators, *N*_1_ = 10^5^, in our heat bath for our other approximations to hold. If these conditions did not hold, we would see that our kernel function has a different form (for example, decaying at a slower rate), and in this case we would have to use a generalized Langevin model as our coarse-graining approach in order to capture the dynamics of the dimer with sufficient accuracy.

**Table 4.** One iteration of the multi-resolution simulation algorithm of the dimer in the heat bath with long-range interactions, where the second monomer is simulated by the Langevin dynamics.

[L1]	Update velocities of the dimer and solvent particles for a half time step using (4.11).
[L2]	Update positions of the dimer and solvent particles using (4.12).
[L3]	Recalculate accelerations of each monomer and solvent oscillators by (4.14)–(4.16).
[L4]	Update velocities of the dimer and solvent particles for a half time step using (4.13).
[L5]	Continue with step [L1] using time *t* = *t* + Δ*t*.

The diffusion constant of the dimer, *D*_*d*_, can again be estimated by numerically integrating the velocity auto-correlation function. Integrating our results from figure 6 over interval [0, 1], we obtain *D*_*d*_ ≈ 0.510, which compares well with the theoretical value, *D*/2 = 0.5, given by equation (2.7).

## 5. Discussion and conclusion

In this paper, we have used two theoretical heat baths. Although these heat baths are based on qualitatively different descriptions of solvent–dimer interactions, they both lead to the Langevin description, given in equations (2.1)–(2.4), in a certain limit. In particular, we can use this limiting process to coarse-grain a part of the simulated dimer molecule, while using a detailed MD model to describe the rest of the molecule. Such a multi-resolution approach has potential to significantly speed up computer simulations of dynamics of macromolecules [38–42], provided that it is combined with additional multiscale and multi-resolution methodologies, discussed below.

Our long-range interaction model leads to the system of generalized Langevin equations, given by equations (4.5) and (4.6). Although we have worked in the parameter regime where the generalized Langevin equations can be well approximated by the system of Langevin equations given by (2.1)–(2.4), this will not be the case in other parameter regimes and for more realistic solvent descriptions, especially when the memory kernel is estimated from MD simulations [58,59]. One possible strategy in this case is to couple a detailed MD model with a stochastic coarse-grained model which is written with the help of additional variables [50–52]. To improve the efficiency of simulations further, one can then coarse-grain such a generalized Langevin description using a Brownian dynamics approach [14,60]. Brownian dynamics modelling can be further coupled with stochastic reaction–diffusion modelling based on lattice-based (compartment-based) methods [22]. Lattice-based models are very attractive for simulations of intracellular processes, because they enable modelling of spatio-temporal processes in the whole cell or its significant parts [61]. Coupling Brownian dynamics with compartment-based approaches has been used in a number of applications, including multi-resolution modelling of actin dynamics in filopodia [62,63] or for modelling intracellular calcium dynamics [64].

In this paper, we have investigated multi-resolution approaches, schematically described in figure 1*a*,*b*. Another class of multi-resolution approaches in the literature considers a fixed subdomain of the computational domain where a detailed modelling approach is used, which is coupled with a coarser model in the rest of the simulation domain [21,22]. Such an approach is useful, for example, when modelling intracellular ion dynamics. Ions pass through an ion channel in single file and an MD model has to be used to accurately compute the discrete, stochastic, current in the channel [65,66], while the details of the behaviour of individual ions are less important away from the channel where copy numbers may be very large. Thus, we can improve efficiency of our simulations if we allow ions to pass between regions with an explicitly modelled heat bath and a region where their trajectories are described by coarser stochastic models [51].

A similar multi-resolution approach can also be designed for our illustrative dimer model. It is schematically shown in figure 1*c*, where we identify the region with explicitly simulated heat bath as $\{x_{1}>b\}=(b,\infty )\times R^{2}$, where *b* is the fixed position of the boundary. We are again interested in the behaviour of the dimer in the MD model which would be considered in the full space, $R^{3}$. However, we now want to replace solvent particles which are in $\{x_{1}<b\}=(-\infty ,b)\times R^{2}$ by a coarser Langevin description (2.1)–(2.4). To do that, we have to carefully consider how we handle the transfer of monomers between {*x*_1_ > *b*} and {*x*_1_ < *b*}. In figure 1*c*, we present a two-dimensional illustration of a monomer when it intersects the interface, {*x*_1_ = *b*}. Such a monomer is subject to the collisions with heat bath particles on the part of its surface which lies in {*x*_1_ > *b*}. This has to be compensated by using a suitable random force from {*x*_1_ < *b*}, so that the overall model is equivalent to (2.1)–(2.4) in the Langevin limit. Such correction terms can be derived analytically for the case of a spherical monomer in our short-range interaction heat bath and are presented in [14,54]. They can be used to couple the MD model with its corresponding Langevin description, which can be further coupled with Brownian dynamics, simulated using a much larger time step [14].

Mathematical analysis of multi-resolution methodologies can make use of the analysis of the model behaviour close to the boundaries of the computational domain. For example, derivations of reactive (Robin) boundary conditions of macroscopic models from their corresponding microscopic descriptions [67–69] can be generalized to the analysis of behaviour of molecules close to hybrid interfaces in multi-resolution schemes [21,30,31]. Analysis of open boundaries of MD schemes (i.e. boundaries which can transfer mass, momentum and energy) can lead to further understanding of multi-resolution schemes such as AdResS and hybrid continuum-particle dynamics [70], which enable efficient simulation of biomolecules at realistic physiological conditions [71].

Equations for coupled detailed/coarse-grained models can be systematically derived using Zwanzig’s projection method, which has been used to address co-existence of atoms and beads (larger coarse-grained units) in the same dynamic simulations [72,73]. The equations of motion take the form of dissipative particle dynamics, which have been coupled with atomistic water simulations to design multi-resolution schemes in the literature [74]. Other multi-resolution methods couple atomistic water with specially designed coarse-grained water models [75] or with a continuum approach [35]. Coupling discrete and continuum approaches can also be done for different molecular species present in the system and our choice of a modelling approach for each species can be based on its relative abundance [76–78].

One of several important points which have been left out from our discussion is the discretization of time. Although our illustrative simulations use the same time step for both the MD model and the Langevin description, this is not the most efficient or desirable strategy, because the MD model requires much smaller time step than the corresponding Langevin equation. There is potential to design more efficient schemes by updating the coarser description only at certain multiples of the time step which is used in the most detailed model [39]. This is also the case when a modeller further coarse-grains the Langevin description into a Brownian dynamics model which uses even large timesteps [14].

## Supplementary Material

Computer Codes for Illustrative Simulations

## Data accessibility

The computer codes used to compute illustrative results in figures 3–6 have been uploaded as part of the electronic supplementary material. Figures 1 and 2 contain no data.
